# In (or outside of) your neck of the woods: laterality in spatial body representation

**DOI:** 10.3389/fpsyg.2014.00123

**Published:** 2014-02-19

**Authors:** Sylvia Hach, Simone Schütz-Bosbach

**Affiliations:** ^1^School of Psychology, The University of AucklandAuckland, New Zealand; ^2^Max-Planck Institute for Human Cognitive and Brain SciencesLeipzig, Germany

**Keywords:** left–right handedness, lateralization, personal space, body representation, somatosensation

## Abstract

Beside language, space is to date the most widely recognized lateralized systems. For example, it has been shown that even mental representations of space and the spatial representation of abstract concepts display lateralized characteristics. For the most part, this body of literature describes space as distal or something outside of the observer or actor. What has been strangely absent in the literature on the whole and specifically in the spatial literature until recently is the most proximal space imaginable – the body. In this review, we will summarize three strands of literature showing laterality in body representations. First, evidence of hemispheric asymmetries in body space in health and, second in body space in disease will be examined. Third, studies pointing to differential contributions of the right and left hemisphere to illusory body (space) will be summarized. Together these studies show hemispheric asymmetries to be evident in body representations at the level of simple somatosensory and proprioceptive representations. We propose a novel working hypothesis, whereby neural systems dedicated to processing action-oriented information about one’s own body space may ontogenetically serve as a template for the perception of the external world.

## INTRODUCTION

Whether we navigate through an unknown environment, cross a busy street or give somebody directions by pointing to a location, for most of us, parts of our right hemisphere will be active (e.g., Kinsbourne and Bemporad, 1984; Corbetta and Shulman, 2002; Craig, 2002; Vogel et al., 2003). Of course this does not mean that the left hemisphere will be silent. Rather, in comparison to tasks which involve more left-hemispheric functions such as language (Grunwald et al., 2001), the spatial processing required during the above examples recruits predominantly right lateralized circuits (Stephan et al., 2007). Besides language, spatial processing is the most widely recognized and best studied lateralized system in the human brain (Gotts et al., 2013).

Lateralization for spatial processing is evident on three levels. First, a behavioral index is provided by the level of success with which spatial tasks are performed by people presumed to be lateralized to a greater degree (i.e., right-handers) compared to people with more bilateral functioning (i.e., mixed-handers and some left-handers; Knecht et al., 2000; Szaflarski et al., 2002). While handedness only constitutes an imperfect proxy for laterality, the general finding is that spatial ability declines with increasing dextrality (Annett, 2002; cf. Gotts et al., 2013). For example, neurologically normal right-handers, but not mixed and left-handers, typically misbisect a line to the left of true center (Jewell and McCourt, 2000; cf. Scarisbrick et al., 1987) – a small spatial bias termed “pseudoneglect” (Bowers and Heilman, 1980). Second, cerebral functioning studies provide evidence of right hemispheric dominance for the processing of spatial tasks (for a meta-analysis, see Vogel et al., 2003) and patients suffering from right hemispheric damage exhibit marked spatial deficits (Kerkhoff, 2001). Finally, a third level of laterality description is that of macro- and microstructural differences between the right and left hemisphere generally (e.g., Thiebaut de Schotten et al., 2011), as well as between the corpus callosum of individuals lateralized to a greater, and those to a lesser degree (e.g., Witelson, 1985; Habib et al., 1991; Jäncke and Steinmetz, 2003; Westerhausen et al., 2004).

Both behavioral and functional studies are most often concerned with visuospatial processing of external space. That is, by far the greatest amount of knowledge on the laterality of spatial processes concerns the visual modality and the perception of the region of space that is within one’s reach (i.e., peripersonal space) and the region of space outside of one’s reach (i.e., extrapersonal space). However, it is also known that lateralization of spatial processing extends to other modalities and even to the mental representation of space and abstract concepts like numbers. Similar to pseudoneglect for the visual variant of the line bisection task, for example, haptic bisection also results in a systematic bias in right-handers (Sampaio and Chokron, 1992; Brodie and Dunn, 2005; Hach and Schütz-Bosbach, 2012) and mental representations of space are frequently affected by a hemispatial bias in patients suffering from visuospatial neglect (Bisiach and Luzzatti, 1978; Bartolomeo et al., 1994; Landi et al., 1997). As a result of the focus on the visual modality, discussions and examinations of representations of our own body, dominated by the tactile and proprioceptive sense, are almost completely absent from the spatial literature. Yet, our own body represents a crucial spatial compartment and, in extension to peri- and extrapersonal space, may be conceptualized as a third region of space. It is this third region of space that forms the main focus of the current review.

## BODY SPACE

Body space is simply defined as the space that our own body inhabits. For the purpose of this review and the working hypothesis we propose as part of the conclusion to this review, body space constitutes a superordinate concept including at least two different body representations that have been proposed to exist in the past. Specifically, the body schema, or a low-level egocentric, action-oriented representation of one’s own body in terms of tactile and proprioceptive information (for various definitions of this concept, see Head and Holmes, 1912; Haggard and Wolpert, 2005; Holmes and Spence, 2006), forms an important part of body space. Similarly, however, the body image may be seen as a part of body space. Body image, here, refers to an abstract representation of one’s own body that includes a conscious and emotional evaluation of the visual characteristics of the body (Paillard, 1999; Gallagher, 2005). This representation is thought to be coherent across space and time and, importantly, distinct from the body schema (Paillard, 1999; Gallagher, 2005; Schwoebel and Coslett, 2005; Dijkerman and De Haan, 2007). In addition to the body schema and the body image, other forms of body representations, such as left-lateralized linguistically mediated representations, have importantly been proposed to exist (e.g., Coslett et al., 2002; Schwoebel and Coslett, 2005; Dijkerman and De Haan, 2007), but are considered distinct from the current conceptualization of body space.

Body space, including the different forms of representations mentioned above, distinguishes itself from peri- and extrapersonal space with regard to two aspects. First, representations of our own body are always immediate, inescapable and tied to the first person perspective. That is, there is no possibility of separating oneself from body space – an observation recorded as early as the late 1800s by James (1890). Second, interoception, or the “sense of the physiological or homeostatic condition” of our own body (Berlucchi and Aglioti, 2010, p. 31), provides a unique and private source of information about the state of this space, that is lacking for the perception of peri- and extrapersonal space. There is extensive evidence showing interactions between body space, peripersonal and extrapersonal space. Specifically it has been shown that the border between what is perceived as within or outside of one’s reach, or neck of the woods, is heavily dependent on the representation of body space (e.g., Pavani and Castiello, 2004; Coello et al., 2008). However, it remains an empirical question whether the aforementioned special characteristics of body space mean that this third region of space can or cannot be subsumed under the heading of spatial perception. In other words, it is unclear whether general rules of spatial perception, specifically those applying to action-oriented egocentric representations of space, also apply to the perception of body space.

One first, and admittedly crude, level of examining this is laterality. Findings showing that body space perception, like that of external space, is largely right-lateralized could be first evidence that, although special with regard to the two points mentioned above, body space can be subsumed under the supramodal heading of spatial perception. In the following, we will summarize the state of evidence of hemispheric asymmetries in body space representation from three perspectives. First, body space in health and second, body space in disease will be examined. Specifically, body space asymmetries in right-handers and two descriptive examples of disorders of body space, namely somatic neglect and eating disorders, will illustrate the crucial contribution of the right hemisphere to these representations. Third, studies pointing to differential contributions of the right hemisphere to illusory body space, using the example of the rubber hand illusion (RHI), will be summarized. Examples from all three levels of description of laterality, namely behavioral, functional, and structural studies will be taken into account.

## BODY SPACE IN HEALTH

Returning to the example of everyday spatial processing given at the outset of this review, the area of navigation has provided some of the best-known literature on spatial processing (e.g., Craig, 2002). Navigational experts like London taxi and bus drivers are widely known to show greater posterior hippocampal volume bilaterally. Right hippocampal volume, in particular, correlates with the number of years of navigational expertise (Wagner et al., 2003; Nico et al., 2010). It is also known that bilateral hippocampal lesions lead to a loss of some of the flexibility with which the navigational expertise acquired prior to injury can be applied (Guardia et al., 2013). Most of these well-known examples focus on navigational skills which require an abstract, view-independent or allocentric representation of external space.

A related line of research examines spatial processing from the egocentric perspective, and has received comparatively little attention. Specifically, studies examining navigational behavior in relation to handedness have shown that healthy right-handers exhibit a behavior similar to stroke victims suffering from neglect. While it is common for right parietal lobe stroke victims to collide with objects to their left when navigating through tight spaces (Tsakiris et al., 2007a; Bekrater-Bodmann et al., 2012), Turnball and McGeorge (1998) presented observational data showing the opposite bias in healthy right-handers. That is, right-handers were found to be more likely to collide with objects on their right. A series of more recent studies has confirmed and extended this finding to show that the extent to which right-handers display a leftward bias on the classical line bisection task is significantly associated with the number of rightward collisions (Nicholls et al., 2007, 2008b) and that navigational displays viewed in the upper, but not the lower, visual field result in a greater chance of rightward collisions (Thomas et al., 2009).

While the authors of the above studies attributed the navigational bias in right-handers only to a biased perception of the display provided (in most studies this is a narrow doorway or corridor), one additional factor that may act together with a biased perception of the peri- and extrapersonal space as well as a host of other factors (e.g., differential movements of the right and left upper limbs, see Nicholls et al., 2007, 2008b) to produce lateralized collisions has been left largely unexplored. It could be argued that just as much as the perception of the external environment, an accurate perception of where in space one’s own body is and how wide/narrow it is in different places is required for this task. Thus, an additional contributing factor to right-handers’ bumping behavior could be a less precise perception of their own body. Due, in part, to the scarcity of validated tests for the assessment of representations of body space, this question has not been addressed in depth until recently.

A few existing examinations of representations of body space employ a task parallel to the traditional line bisection task. Here participants are required to point to their body midline or to a location ahead of them which corresponds with their body midline (also termed subjective sagittal middle). Three studies examined handedness differences in this task and report findings broadly congruent with pseudoneglect. Spidalieri and Sgolastra (1997) and Chokron et al. (2004) reported that right-handed participants pointed significantly to the left of their midsagittal plane when using their left hand, while another study found a significant bias to the left for both right- and left-handers (Colliot et al., 2002). Using this task it is not possible, however, to distinguish between a bias affecting pointing actions and a spatial bias which affects the representation of one’s own body. Further, a midsagittal pointing task could be argued to draw less on a spatial representation of the body as a whole.

A different task which is superior in that it requires many body surface locations to be transformed into locations in external space (cf. Yamamoto and Kitazawa, 2001) is the Fluff Test. Here, a number of cotton balls are attached to the blindfolded participant’s clothes. Subsequently, the participant is required to recover these. Using this task, first evidence of a body space bias in neurologically healthy right-handers beyond a pointing bias such as that documented using the midsagittal pointing task was found by Cocchini et al. (2001). For their validation of the Fluff Test, which was aimed at assessing somatic neglect in stroke patients, the authors also examined the performance of a group of control participants. A trend toward handedness differences was found with some left-handers outperforming right-handers.

A more recent study conducted at our lab improved the sensitivity of the Fluff Test by increasing the number of items to be recovered from the body surface (Hach and Schütz-Bosbach, 2010). By using a tight-fitting full body suit equipped with the stimuli that were to be recovered, some limitations such as the potential of tactile feedback during the placement of the cotton balls on the clothing of the participant were also removed. We confirmed the original trend and showed that right-handers generally showed less exploration of their body surface in comparison to left-handers. In line with studies reporting handedness differences for cognitive domains as diverse as attention, decision making or memory (e.g., Annett, 2002; Propper et al., 2005; Christman et al., 2007b), we interpreted this as an indication of right-handers having less access to right hemisphere processing. Functional access in this case refers to the recruitment of specialized neural structures for performance on a task that is high on the demands of this particular ability and “better functional access” results from greater neural interconnectivity (higher number of white matter tracts/synapses; He et al., 2007). Further, when biomechanical constraints were taken into account, a particular advantage for the right (or disadvantage for the left) side of the body seemed to be present for this group of participants.

Moreover, we also introduced a quantitative measure of the putative body space bias, the Body Outline Pointing Task (Hach and Schütz-Bosbach, 2010). This measure requires participants to point to the widest and narrowest part of their hidden body on their right and their left side in three locations. A comparison of participants’ pointing scores for the left and right hemispace can be used to determine a side bias. In addition, a more precise measure of participants’ ability to judge the *actual* spatial dimensions of their own body is possible by utilizing measurements taken from a standardized photograph of the participant. We found right-handers to show a spatial asymmetry with respect to the distance from the midsagittal plane in two out of the three locations when comparing left- and rightward pointing movements. Their estimate of the narrowest part of their right waist as well as of the widest part of their right hip was found to be more distant from the midsagittal plane and importantly closer to their actual body boundary compared to their estimate of the left waist and hip. Significant correlations between performance on this task and individual participants’ laterality quotients further showed that performance decreased with increasing dextrality.

Other work showing evidence consistent with the conclusion that right-handers represent body space less precisely than left-handers includes that of Linkenauger et al. (2009). According to their findings, right-handers significantly overestimate the length of their right arm and the size of their right hand while left-handers show no such asymmetry. There is also some evidence of greater areas of somatosensory cortex devoted to the representation of the right hand in right-handers (Sörös et al., 1999). However, other studies have failed to replicate this finding (e.g., White et al., 1997; Jung et al., 2003). Nevertheless, there is the possibility that in addition to less access to, or an overall less precise, structural representation of the body in right-handers, there may be more “hardware” devoted to the representation of the dominant side of the body causing right-handers to show some asymmetry in judging spatial properties of their own body.

**FIGURE 1:**
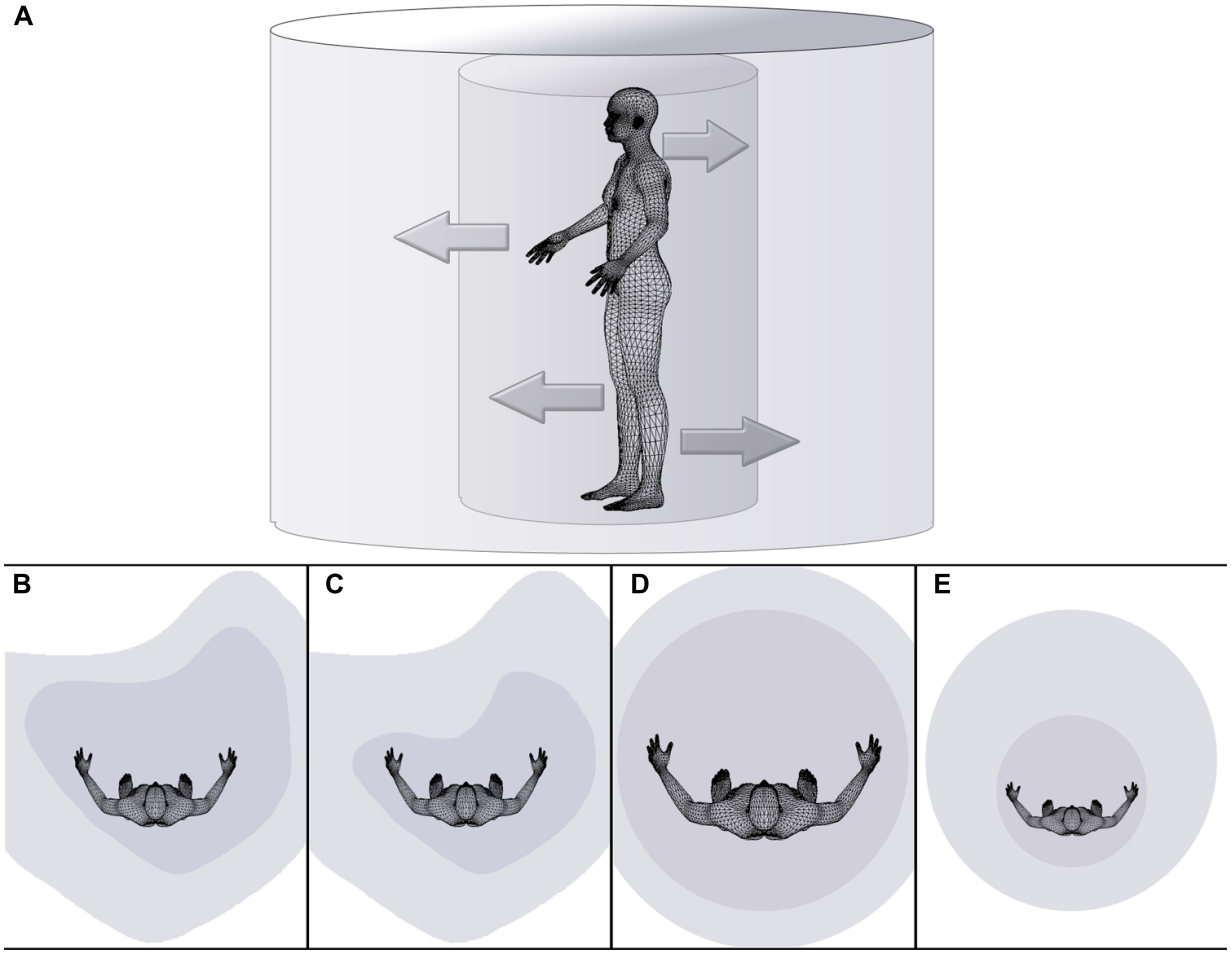
**Schematic drawing illustrating the dependency of the extent of peripersonal and extrapersonal space on body space in health (A,B), disease (C,D), and during a body illusion (E). (A)** The inner cylinder represents peripersonal space, while the external cylinder symbolically represents extrapersonal space. The inner arrows symbolize the dependency of the extent or size of peripersonal space (i.e., the internal cylinder) on the state of body (space), while outer arrows pointing from the inner to the outer cylinder symbolize the dependency of the extent or size of extrapersonal space on the state/size of peripersonal space. Distorted space perception is illustrated for healthy right-handers **(B)**, individuals affected by hemispatial neglect **(C)**, individuals effected by eating disorders on the example of anorexia nervosa **(D)**, and individuals experiencing a small body illusion (e.g., van der Hoort et al., 2011; Banakou et al., 2013) **(E)**.

To summarize the literature reviewed thus far, there is first evidence of laterality effects in the spatial representation of the body of healthy individuals. Right-handers show a bias not only with respect to judging external space but also with judging the spatial characteristics of their own body (see **Figure 1B**), and this bias is absent in the performance of left-handers. Moreover, the reported effects relate to an action-oriented moment-to-moment representation of the spatial properties of our body akin to the body schema, which may be supported by automatic sensorimotor loops independent of explicit awareness (Paillard et al., 1983; Paillard, 1999; Rossetti et al., 2005; Gallace and Spence, 2008).

## BODY SPACE IN DISEASE

One condition previously mentioned in relation to the mental representation of space and the spatial representation of abstract concepts as well as in relation to dysfunctional navigation behavior is that of hemineglect. Hemineglect probably constitutes the most striking disorder which can result from disruptive perfusion from the middle cerebral artery. Most commonly, hemineglect results from right hemispheric infarcts (Bisiach and Vallar, 2000; Kerkhoff, 2001), although sporadic reports of left hemispheric origin also exist (e.g., Peru and Pinna, 1997). In the absence of sensory deficits, individuals with hemineglect display a decreased propensity to recognize and act on objects located contralesionally, although implicit recognition may be preserved (Brozzoli et al., 2006). While most studies concentrate on peri- and extrapersonal space deficits, deficits in the representation of the stroke patient’s own body have also been reported (Bisiach et al., 1986; Committeri et al., 2007). In other words, in addition to a deficit in perceiving extrapersonal space (see **Figure 1C**), a diminished perception of the contralesional body half, or somatic neglect, is present in some cases. First reports of disturbances of this nature date back as far as the early 1900s (Head and Holmes, 1912; Pick, 1922) but few in-depth reports of this condition have emerged since.

Somatic neglect describes a complete disregard of the contralesional side of the patient’s body. For example, these patients may comb their hair, shave or dress only the non-affected right side of their body (e.g., Bisiach et al., 1986). Measures aimed at detecting and quantifying these deficits are structured in a similar way in that they require the patients to perform reaching movements for their contralesional extremity or the contralesional side of their body (Reaching Task: Bisiach et al., 1986; Fluff Test: Cocchini et al., 2001; Comb, Razor and Glasses Test: Zoccolotti and Judica, 1991). When asked to complete the subjective midsagittal task described above, a lateral translation to the right is frequently observed (Richard et al., 2000, 2004a,b; Saj et al., 2006). Overall, however, there has been a paucity of sensitive tasks, and existing measures are mostly not part of routine assessment following admission to hospital with cerebral infarction. For this reason, it is difficult to draw conclusions about important questions such as the frequency with which somatic neglect occurs, the extent to which a decrease in the awareness of one’s own body space is typically shown, the duration of this deficit and what impact it has on the patient’s daily life.

There is also inconclusive literature regarding the association between somatic or personal neglect and extrapersonal hemineglect. That is, a decrease in the awareness of one’s own contralesional hemibody may or may not be associated with neglect of peripersonal and extrapersonal space (Bisiach et al., 1986; Zoccolotti and Judica, 1991; Beschin and Robertson, 1997; Ortigue et al., 2006; Committeri et al., 2007). On the whole it appears that more instances of isolated extrapersonal neglect than instances of pure somatic neglect have been found, however. The contradictory state of the literature may, in part, result from the use of different measures of somatic neglect in different studies (Guariglia and Antonucci, 1992), the different levels of severity of deficit examined or the varying temporal intervals between infarct and assessment.

Similarly, to date only two studies have examined the effectiveness of prism adaptation on the extent to which somatic neglect signs are shown, and their results contradict each other. As part of this intervention, patients wear prism glasses, which induce an optical shift. Following an initial adaptation phase during which pointing movements are performed in a spatially shifted manner, the oculomotor system resets itself and the patient’s movements will be adjusted for the optical shift (Luaute et al., 2006a,b). Short-term use of prisms has been found beneficial in the treatment of hemineglect, in the amelioration of representational neglect (e.g., spatial representation of time; Magnani et al., 2011) as well as spatial deficits shown in other modalities including audition (Buxbaum et al., 2004; Hamilton et al., 2008; Jacquin-Curtois et al., 2010). In a detailed study of the effect of prism adaptation on different aspects of somatic neglect, an improvement of tactile performance was found, which resulted in a significant decline in somatic neglect symptoms on clinical assessment (Serino et al., 2007). This contrasted with no beneficial effect for proprioceptive and motor symptoms for the same patients. A smaller, more recent study failed to show any long-term effect of prism adaptation on personal space representations (Rusconi and Carelli, 2012), although follow-up reports are necessary, as the study sample of this preliminary report only comprised three patients.

Turning from the behavioral level of description to the functional level, it was noted earlier that traditionally right middle cerebral artery infarcts have been associated with hemispatial deficits. Crucially, areas in the vascular territory of the right middle cerebral artery not only support processing of peri- and extrapersonal space (Pouget and Driver, 2000; Driver and Vuilleumier, 2001; Husain and Nachev, 2006), but also the representation of personal or body space (Kinsbourne and Bemporad, 1984; Colby and Duhamel, 1996; Graziano and Cooke, 2006). On measures of somatic neglect, cortical areas as disparate as the supramarginal and post-central gyrus as well as the medial white matter (Committeri et al., 2007) and the superior temporal gyrus (Karnath et al., 2004, 2011) may be involved. In addition, personal neglect may be the result of a functional disconnection between primary regions for coding proprioceptive and somatosensory input and regions coding a more abstract spatial representation of the body (Committeri et al., 2007).

In line with some of these findings, studies that investigate patients with conditions related to somatic neglect, such as autotopagnosia, suggest a key role of the right inferior parietal lobe (e.g., Ogden, 1985; Sirigu et al., 1991; Buxbaum and Coslett, 2001). Autotopagnosic patients display a deficit in maintaining spatial relationships of body parts and make mislocalization errors when asked to point to specific body parts. Hemianesthesia has also been reported to occur more frequently following right brain damage (Sterzi et al., 1993) and anosognosia, a lack of conscious awareness of a deficit often concomitant to hemineglect, is associated with right parieto-temporal lesions (Orfei et al., 2007). Finally, somatoparaphrenia, a condition where ownership of individual limbs is consistently ascribed to somebody else, is typically associated with right hemispheric damage (Bottini et al., 2002; Vallar and Ronchi, 2009).

These latter studies are important in providing robust evidence regarding well-circumscribed areas of the brain, which support representations of one’s own body. Valuable insight into more theoretical questions can also be gained from experimental studies of individual patients suffering from any of these conditions. A nice example of this is a study by Fotopoulou et al. (2011) which examined the performance of two somatoparaphrenic patients before and after mirror box therapy. Therapy was successful in transiently reinstating limb ownership when a third person perspective was experimentally induced, but this was not accompanied by an improvement of somatoparaphrenia symptoms beyond the experimental session. Both patients showed extensive damage encompassing most of the right parietal and temporal lobes, which does not allow any precise conclusions about the exact networks supporting limb ownership. However, the results crucially suggest that body ownership is largely driven by an egocentric representation of the body (Blanke et al., 2004; Tsakiris et al., 2007b; Petkova et al., 2011).

Relatedly, it has been proposed that allocentric, or viewer-invariant, neglect always occurs concomitant to egocentric neglect (Grunwald et al., 2001; Rorden et al., 2012). That is, aside from the question of which regions of space are affected separately or in combination by hemineglect (i.e., somatic neglect with or without extrapersonal neglect), patients showing deficits in recognizing target objects on the left side of the page (egocentric neglect) often also show deficits in recognizing individual targets if the defining feature is on the left side of the target object, regardless of the location in which it is presented in egocentric coordinates (allocentric or object-based neglect). Although this may depend on the exact nature of the search task and the characteristics of the stimuli (see Gainotti and Ciaraffa, 2013 for a dissociative account of ego- and allocentric neglect), it may be deduced that the ability to recognize and process external objects as a whole from one’s own perspective contributes to a representation of the same object in a manner that is separate from that perspective. Similarly, the representation of our own body from our perspective is the most immediate and private representation of space, and this representation may enable us to not only construct an allocentric model of our own body, but also a model of space outside of our own body (see **Figure 1A**). As shown above, higher-order deficits, such as extrapersonal neglect or somatoparaphrenia, can frequently result from a difficulty in synthesizing the lower-order moment-to-moment representation of one’s own body with the representation of external spatial cues as well as the visual/external cues about the spatial properties of one’s own body.

A second example of a well-known set of disorders which affect the spatial representation of one’s own body is that of eating disorders. In contrast to somatic neglect and some of the related syndromes described above, it is usually assumed that, in eating disorders, the body image is affected (e.g., Kinsbourne and Bemporad, 1984). As a result of a negative evaluation of their own body, individuals with eating disorders go to extreme lengths to alter the appearance of their body. Disturbances of the body image are most frequently assessed using questionnaires and tests that tap into the conscious and emotional evaluation of body space. For example, Hennighausen and Remschmidt’s (1999) Computer Body Image Test contains partly distorted individual body outlines, generated by the tracing of a photograph of the patient’s body. The patient is then asked to adjust the body outline according to the estimated true size of his or her body outline. This test can be regarded as an explicit measure of how body space is represented in the sense that it requires a memory-based recall of the spatial dimensions of one’s own body that is independent of direct primary somatosensory inputs due to movement.

Nonetheless, the body image also constitutes a kind of structural and geometric representation of the body. That is, the body image is principally dependent on and influenced by primary somatosensory inputs (cf. e.g., Lackner, 1988; Gandevia and Phegan, 1999; Schütz-Bosbach et al., 2009a). This is especially true during development. More recent investigations of participants with a history of eating disorders have begun to acknowledge this interdependency and include tests of primary sensory and motor representations of the body. As a result, it is now known that individuals with anorexia nervosa perform more poorly on tests requiring the integration of primary sensory input with external spatial reference frames. Marked deficits have been reported for the haptic reproduction of random shapes (Grunwald et al., 2001) and for the alignment of the subjective vertical with the external reference frame (Guardia et al., 2013), for instance.

Individuals with anorexia nervosa also show deficits in tasks assessing their ability to perform motor imagery. In a study by Guardia et al. (2012), anorexia nervosa patients and a group of control participants were required to perform a task similar to the navigation and handedness studies summarized above. A navigational display containing a doorway of varying size was presented. Instead of navigating through the doorway, participants remained at a fixed distance to the display and had to judge whether or not they could fit through without turning sideways (first-person condition). In an additional condition, the same judgment had to be made about the experimenter standing in the same position as the participant for the first-person judgment (third-person condition). There was a significant difference between first- and third-person perspectives only for participants with a history of anorexia nervosa. These patients overestimated the dimensions of their own, but not the experimenter’s body, relative to the doorway. Importantly, this difference was not due to decreased perceptual discriminability in the patient group.

These findings are consistent with a number of other reports showing a lesser ability of anorexic participants to directly, or indirectly, estimate their body boundaries (see **Figure 1D**). For example, Christman et al. (2007a; see also Niebauer et al., 2002) found a significant correlation between the absolute value of handedness and the discrepancy between actual and estimated body mass index (BMI): the greater the degree of right-handedness, the greater the discrepancy between the true and estimated BMI. The authors suggested that greater lateralization in right-handers leads to diminished access to right hemisphere processing and, as a result, to an impoverished representation of the body. Similarly, in an indirect measure of body boundaries, Nico et al. (2010) found anorexic patients to be less accurate in their estimation of the width of their left upper body. Crucially, performance of anorexia nervosa patients was comparable to that of a group of participants with a history of right parietal lesions.

Finally, a few studies have investigated body representation in individuals with eating disorders with the use of the RHI paradigm. A summary of the experimental procedure typically employed for this illusion will be given below. For the moment, it is sufficient to say that the illusion critically involves a comparison of the experimentally manipulated tactile and visual information with the proprioceptive information about the position of one’s own hand. Since the representation of the hand is not usually a focus of body concerns or emotional biases, this illusion is arguably better-suited to the study of body awareness in individuals with eating disorders compared to any of the paradigms mentioned above (Schütz-Bosbach et al., 2009b). Eshkevari et al. (2012) and, prior to that, Mussap and Salton (2006) found a relationship between the extent of psychopathology and the strength of the illusion. Specifically for the former, nearly a quarter of the variance in the strength with which the illusion was felt was explained by interoceptive deficits and self-objectification. Participants with increased scores on interoceptive items (e.g., “I don’t know what is going on inside me.”) of the Eating Disorder Inventory and increased scores on a self-report assessment of self-objectification showed a greater behavioral effect of the illusion (i.e., greater proprioceptive drift). In sum, studies examining the susceptibility of individuals to the RHI are consistent with the theory of right hemispheric dysregulation and resulting body spatial deficits in individuals with eating disorders.

## ILLUSORY BODY SPACE

The use of illusory paradigms to study body awareness has a long tradition, particularly in the literature examining neurologically healthy individuals. These studies typically create a mismatch or dissonance between the sensory modalities, and capture participants’ responses both on a qualitative or subjective level (with the use of questionnaires) and a quantitative level (an objectively measurable effect such as the displacement of a body part). Somatosensory illusions provide an excellent demonstration of the malleability of the boundary between external and body space. In other words, what is experienced as embodied space at one moment becomes disembodied as a result of the illusion being induced a moment later (see also Holmes and Spence, 2006 for the concept of excorporation). And the reverse is true as well, with external space transforming into embodied space (i.e., incorporation).

The RHI constitutes one of the most widely used examples of perceptual illusions employed to study aspects of body representation, body awareness, and body ownership. The typical set-up includes a screen which conceals one of the participant’s hands and forearm from their view, a prosthetic (rubber) hand, which is placed in an anatomically plausible position to the participant’s body (cf. Armel and Ramachandran, 2003; Tsakiris and Haggard, 2005) and a set of stroking devices. In a block-wise fashion, the participant’s concealed hand and the prosthetic hand are stroked in a synchronous or asynchronous manner. While synchronous stroking induces a displacement of the tactile stimulation toward the location of the prosthetic hand (i.e., the illusion of the tactile sensation originating from the seen rather than the felt position), asynchronous stroking does not usually produce such a displacement. Both horizontal and vertical experimental set-ups have been used to successfully evoke displacement in the left–right and up–down direction, respectively (Haggard and Jundi, 2009; Bekrater-Bodmann et al., 2012). Participants’ responses are measured with regard to the phenomenological experience of ownership over the prosthetic hand (Longo et al., 2008), and with respect to sensory aspects of the illusion such as the degree of displacement (also commonly termed proprioceptive drift).

Perhaps unsurprisingly given the spatial nature of the illusion and the involvement of representations of the body, it has repeatedly been shown that extensive right hemispheric networks appear to support the illusion. Converging evidence from functional magnetic resonance imaging (fMRI), transcranial magnetic stimulation (TMS), and lesion studies point toward the temporo-parietal junction (Tsakiris et al., 2008), inferior parietal lobule (Kammers et al., 2008), posterior insula (Tsakiris et al., 2007a), and ventral premotor and cerebellar areas (Ehrsson et al., 2004, 2005; Zeller et al., 2011) as contributing to some aspects of the illusion.

While it may be said that due to their exclusive focus on representations of the hand, RHI findings are limited in the conclusions that can be drawn about representations of the body as a whole, other studies have successfully created the illusion of incorporating a foreign body using a similar tactile stimulation procedure (Lenggenhager et al., 2007, 2009; Petkova and Ehrsson, 2008). It is also known that there are varying degrees to which participants perceive this type of illusion and a certain percentage of people from the general population (also called “non-perceivers” in contrast to “perceivers”) appear to be somewhat immune to the illusion. Research into individual differences in susceptibility to this and other somatosensory illusions is only starting to emerge, but it is this work which may be particularly interesting with regard to the laterality aspect of the illusion.

One of the first studies to examine individual differences in the RHI is that by Niebauer et al. (2002). The authors compared the susceptibility of strong right-handers with that of less strongly right-handed participants. Remarkably, it was found that the latter group reported a stronger experience and a tendency to a faster onset of the illusionary experience of incorporating a prosthetic hand into their body schema. The authors proposed that due to the assumed greater right hemisphere access, the less strongly handed were more “efficiently” able to update their body representation and thus experience the illusion to a greater extent.

Another recent exception to the lack of studies examining individual differences contributing to the extent to which sensory illusions are experienced is that by Tsakiris et al. (2011). The objective of this study was to determine the extent to which interoceptive abilities affect the integration of multiple sources of sensory information about the body. Similar to the studies examining participants with eating disorders cited above, Tsakiris et al. (2011) found that low interoceptive ability was associated with a stronger illusion generally and, more specifically, with greater proprioceptive drift, greater reduction in skin temperature of participant’s own hand and greater feelings of ownership over the rubber hand. The authors conclude that the differential weighting of internal and external sources of information about the state of the body underlies the difference in susceptibility to the RHI. Greater weighting of right-hemispheric internal signals (Craig, 2002) may lead to a decrease in the extent to which the illusion is perceived. Unfortunately, no information about the laterality of the participants included in this study was given, and, at present, there are no investigations of the relationship between the degree of lateralization and interoceptive ability.

Finally, a study by Ocklenburg et al. (2010, p. 180) showed greater skin conductance response to a threat to the left hand as well as “a stronger subjective identification with the rubber hand on the left side” following the induction of the RHI compared to the response of the right hand. Interestingly, more recent work by the same group has shown that individuals affected by chronic regional pain syndrome, a condition characterized by unilateral deficient perception of static tactile stimuli, show a similar laterality effect (Reinersmann et al., 2013). Left-affected individuals reported a stronger RHI on their left hand compared to right-affected individuals. Furthermore, a significant negative correlation between the time passed since the onset of the disease and the strength of the illusion was only found for left-affected individuals. However, there is substantial clinical heterogeneity in chronic regional pain syndrome and bilateral cortical reorganization has also been reported (Marinus et al., 2011). Therefore, these latter findings will need to be interpreted cautiously.

Consistent with Ocklenburg et al.’s (2011) finding of an enhanced RHI for the left hand, it has also been found that neurologically healthy right-handers are particularly receptive to spontaneous sensations for their left hand compared to their right hand (Michael et al., 2012). In this last study, there was a significant difference between the left and right hand in the number of different spontaneous sensations (e.g., beat/pulse, tickle) reported after a ten second block as well as their intensity and spatial extent. Furthermore, visual attention modulated the effect spatially, by shifting the spontaneous sensations more distally (i.e., from the palm to the fingertips). Together the RHI and the spontaneous sensation finding suggest that the right hemisphere gives rise to a representation of the sensations arising from the left body half that is updated at a higher rate compared to tactile and visual signals from the right body half arriving at the left hemisphere. Both the weighting of internal to external signals within these representations of the right and left body half, and the weighting of right and left hemispheric contributions to the moment-to-moment representation of the body on the whole may contribute to individual differences in susceptibility to illusory percepts such as the RHI.

In summary, perceptual illusions pertaining to representations of the body are important in supplementing findings from the clinical literature because they enable the examination of intact systems in the healthy brain and body. They further illustrate the flexible boundary between what is perceived as part of our own body and that which is perceived as outside of our own body by inducing illusory shifts causing incorporation of space outside of the body and excorporation of body space. The RHI is thus inherently spatial, acting on body location. A growing body of research shows that right hemispheric networks support the induction of the RHI and that individuals with a lower degree of lateralization are more susceptible to the illusion. Further, there is some evidence that individuals with low interoceptive abilities may experience the RHI more easily and vividly.

## CONCLUSION

An ever-increasing amount of literature documents laterality effects with regard to space in vision (e.g., Brodie and Dunn, 2005; Cocchini et al., 2007), audition (e.g., Ocklenburg et al., 2010), the mental representation of numbers and the alphabet (e.g., Oliveri et al., 2004; Nicholls et al., 2008a) and even seemingly mundane tasks including Likert scale responses (Nicholls et al., 2006). Even though the hemispatial bias resulting from lateralization is small in magnitude in any one of these instances, its omnipresence means that the consequences are far-reaching. While external spatial representation has long been recognized as a lateralized system and many theories have been proposed as to the mechanism by which this division of function occurred (for some examples, see Davidson and Hugdahl, 2002), the spatial representation of abstract concepts and spatial representations created by modalities other than vision have only recently warranted attention.

Here, we summarized evidence from three different areas of body space literature which shows that laterality is a principle that governs the multimodal processing of this region of space also. Specifically, first evidence of right-hemispheric dominance for spatial body representations was shown to exist for simple, primary sensory representations of body space such as those utilized during pointing movements to one’s own body and those at play in body illusions like the RHI, as well as more complex spatial representations of one’s own body such as the distorted body space in individuals with eating disorders. It appears, therefore, that despite constituting a richer and more immediate spatial representation through the additional interoceptive component and the inescapable first-person perspective, body space is equivalent to external space in the sense of being supported by right-lateralized networks.

From the above observations we conclude the present review by proposing a working hypothesis stating that rather than many different spatial systems for different purposes, one main system may exist which allows for the action-oriented representation of one’s own body and the parts of the external spatial world on which the action could potentially be applied. In other words, it may be most parsimonious and computationally efficient (see Gotts et al., 2013 for a summary of a similar argument regarding a computational benefit of lateralization) to possess one system for the processing of spatial information as a whole, be it for navigating from one side of the room to the other and avoiding collisions between one’s own body and objects in the path, estimating the dimensions of a cup in relation to the size of one’s own hand in order to grasp it or reaching for an itch on the back of the neck. Furthermore, it could be argued that due to the immediacy of spatial representations of one’s own body, it is this representation that forms the vantage point for the perception of the world. In other words, not only might the size of one’s neck determine what is perceived as in or outside of the wood, but the spatial representation of our own body may serve as the template for representing external (peri- and extrapersonal) space (see **Figure 1A**).

A related hypothesis commonly summarized under the heading of “embodied perception” similarly emphasizes the interdependency between the perception of the external world and the state of the perceiver’s body (for a summary of prominent accounts, see, for example, Sebanz and Knoblich, 2010). For example, a widely cited piece of empirical evidence for this hypothesis is that the slope of a hill is apparently estimated as steeper by a metabolically challenged perceiver (who may be tired or may carry a heavy weight on their back) compared to a perceiver in a metabolically “neutral” state (Bhalla and Proffitt, 1999). While this account is complementary to the hypothesis forwarded as part of the present review, specifically with regard to metric representations of the body and their influence on the representation of the size of the environment (e.g., Linkenauger et al., 2011), two main differences exist. First, most theorizing that may be subsumed under the heading of embodied perception does not include a discussion of what the body itself constitutes (Proffitt and Linkenauger, 2013). Here, we conceptualize the body as a region of space and propose that the processing of this space may underlie similar principles as the processing of external space. Second, as evident from the example given above, the majority of the embodied perception literature is concerned with the influence of (the state of) the body on the visual perception of the world (e.g., Proffitt, 2013). We, in contrast, emphasize the importance of multisensory cues in the perception of body space and external space.

Support for the hypothesis of body space acting as a template for the action-oriented perception of external space can be found in the developmental trajectory of body and external spatial awareness, or the order in which these functions are acquired. For example, while children as young as 5 months can differentiate between the spatial pattern of self-produced movement and movement produced by another child (Schmuckler and Fairhall, 2001), depth perception and allocentric spatial encoding do not develop until children reach the toddling stage (e.g., Kermoian and Campos, 1988). Children who experience a developmental delay also often display deficits in spatial orientation, but no such deficits are present for the spatial representation of their own body (Herman and Siegel, 1978). Finally, (developmental) Gerstmann’s syndrome is characterized not only by finger agnosia, but also by left–right disorientation and acalculia (Vallar, 2007; Rusconi et al., 2010) and may represent an instance where a deficit in the egocentric spatial representation (i.e., one’s own hands) leads to difficulties in higher-order spatial representations (i.e., numbers) through the process of counting by using one’s own hands, for example.

Returning to the examples of body space lateralization given in the preceding review, findings from the literature on hemispatial neglect are also congruent with the conjecture advanced here. As outlined above, hemispatial deficits of personal and extrapersonal space often occur together (e.g., Rorden et al., 2012). Importantly, isolated personal space deficits are rare, while instances of isolated deficits in extrapersonal space have been reported more often. The example of distorted space in individuals with eating disorders may similarly be interpreted as evidence of the inter-relatedness of personal and external spatial representations, where distortions in the egocentric representation of body space may result in external spatial deficits downstream (see **Figure 1D**). Finally, body space illusions, such as the RHI and related phenomena, most strikingly the embodiment of a whole body of the other gender (Petkova et al., 2011) and of a child’s body (Banakou et al., 2013), nicely illustrate the automaticity and impenetrability with which self-attribution takes place (Jeannerod, 2003). In the case of visual, tactile, and proprioceptive information arriving from roughly the same location in space, it appears that the representation of one’s own body is adjusted to that location, and importantly, representations of external space are calibrated to it. For example, external objects appear to shrink in the face of an embodied large hand (Haggard and Jundi, 2009) or to grow in the face of illusory ownership over a small body (van der Hoort et al., 2011; Banakou et al., 2013; see **Figure 1E** for a schematic illustration of the scaling of peri- and extrapersonal space following the illusory embodiment of a small body).

In sum, we show hemispheric asymmetries to be evident in body representations at increasing levels of complexity from simple somatosensory and proprioceptive representations to higher-order representations. It should be noted that the examples of anorexia nervosa and the RHI reviewed here were selected due to the relatively high number of empirical studies examining these with regard to laterality. Many other conditions which may serve as examples of changes in body spatial representations (e.g., body dysmorphic disorder) could be equally informative here but were outside of the scope of this review. Based on findings from developmental, clinical, and experimental neuroscience, we propose the working hypothesis that spatial representations of one’s own body may not only determine what is within one’s reach or within one’s neck of the woods, but serve as a basis for the action-oriented spatial perception of peri- and extrapersonal space. Related to higher cognitive functions, this may be interpreted more broadly as representations of the bodily self-constituting a template for representations of the external world.

## Conflict of Interest Statement

The authors declare that the research was conducted in the absence of any commercial or financial relationships that could be construed as a potential conflict of interest.
